# Effect of Methionine Supplementation on Rumen Microbiota, Fermentation, and Amino Acid Metabolism in In Vitro Cultures Containing Nitrate

**DOI:** 10.3390/microorganisms9081717

**Published:** 2021-08-12

**Authors:** Faiz-ul Hassan, Yanxia Guo, Mengwei Li, Zhenhua Tang, Lijuan Peng, Xin Liang, Chengjian Yang

**Affiliations:** 1 Key Laboratory of Buffalo Genetics, Breeding and Reproduction Technology, Ministry of Agriculture and Guangxi Buffalo Research Institute, Chinese Academy of Agricultural Sciences, Nanning 530001, China; f.hassan@uaf.edu.pk (F.-u.H.); gyxlq0417@163.com (Y.G.); lmw1607@163.com (M.L.); tangzhyaya@163.com (Z.T.); lijuanpeng2000@163.com (L.P.); liangxinbri@163.com (X.L.); 2 Institute of Animal and Dairy Sciences, Faculty of Animal Husbandry, University of Agriculture, Faisalabad 38040, Pakistan

**Keywords:** methionine, rumen microbiota, methanogenesis, amino acid metabolism, buffalo

## Abstract

This study evaluated the effect of methionine on in vitro methane (CH_4_) production, rumen fermentation, amino acid (AA) metabolism, and rumen microbiota in a low protein diet. We evaluated three levels of methionine (M0, 0%; M1, 0.28%; and M2, 1.12%) of in the presence of sodium nitrate (1%) in a diet containing elephant grass (90%) and concentrate (10%). We used an in vitro batch culture technique by using rumen fluid from cannulated buffaloes. Total gas and CH_4_ production were measured in each fermentation bottle at 3, 6, 9, 12, 24, 48, 72 h of incubation. Results revealed that M0 decreased (*p* < 0.001) the total gas and CH_4_ production, but methionine exhibited no effect on these parameters. M0 decreased (*p* < 0.05) the individual and total volatile fatty acids (VFAs), while increasing (*p* < 0.05) the ruminal pH, acetate to propionate ratio, and microbial protein content. Methionine did not affect ruminal AA contents except asparagine, which substantially increased (*p* = 0.003). M2 increased the protozoa counts, but both M0 and M1 decreased (*p* < 0.05) the relative abundance of Firmicutes while increasing (*p* < 0.05) the Campilobacterota and Proteobacteria. However, *Prevotella* and *γ-Proteobacteria* were identified as biomarkers in the nitrate group. Our findings indicate that methionine can increase ruminal asparagine content and the population of *Compylobactor*.

## 1. Introduction

Enteric fermentation in the rumen leads to methane (CH_4_) production, which contributes to the overall greenhouse gas (GHG) emissions and results in significant dietary energy losses. Due to its adverse consequences, controlling rumen methanogenesis is envisaged as an opportunity to reduce GHG emissions and improve the feed efficiency in ruminants [1]. Maximizing the flow of metabolic hydrogen ([H]) in the rumen away from CH_4_ and toward VFAs would increase the efficiency of ruminant production and decrease its environmental impact. Czerkawski [2] proposed that inhibiting methanogenesis could favor microbial biomass production as an alternative [H] sink. Similarly, Ref. [3] suggested that [H] incorporated into excess NADH was redirected to fatty acid synthesis and fermentation end products such as lactate and ethanol, although the latter sinks were not quantitatively important [1].

Various strategies to control CH_4_ formation in the rumen are currently being investigated. Rumen methanogenesis can be strongly inhibited by various chemical compounds [4] and oils such as linseed oil [5]. While some of these additives and ingredients can inhibit CH_4_ production effectively, benefits in production have been inconsistent despite the theoretical gain of energy not lost as CH_4_ [6]. Meta-analysis of in vitro evidence from multiple experiments has shown some undesirable and incompletely understood consequences of inhibiting methanogenesis, such as a decrease in total enthalpy output in VFA and a consistent decrease in the recovery of reducing equivalents pairs ([2H]) recovered in the main fermentation products [1]. It would be important to gain a thorough understanding of the changes occurring in [H] sinks when methanogenesis is inhibited in rumen fermentation. Hydrogen is required for methane production and affects the biohydrogenation of fatty acids, which is the main metabolic activity of rumen microorganisms. Both methanogenesis and fatty acid biohydrogenation require the participation of rumen microbes and hydrogen, and there is a mutual relationship between them. Any change in the fermentation mode involving the reduction of methane production may affect the whole fatty acid metabolism, including the fatty acid biohydrogenation pathway. As nitrate is a major hydrogen consuming compound, it is thus important to investigate its effect on [2H] production in the rumen [7].

Protein nutrition of dairy cows in recent years has shifted from crude protein towards addressing the ammonia and AA needs for ruminal fermentation to maximize microbial protein (MCP) synthesis [8]. If the AA requirements of rumen microbes could be met, total dietary protein could be lowered without adverse effects on production. Methionine, as a sulfur containing AA, is proteogenic, along with cysteine [9]. Methionine is a limiting amino acid for ruminants and also used as a precursor for CH_4_ production. Both nitrate and CH_4_ can be used as N resources; therefore, methionine is associated with amino acid metabolism. Hence, it is worth investigating how the supplementation of methionine in the presence of anti-methanogenic agents (like nitrate) affects the N and amino acid metabolism, which would help us to devise better nutritional interventions to enhance nutrient efficiency while reducing CH_4_ emissions. Dietary nitrate has shown to effectively decrease CH_4_ production under in vitro [10,11] and in vivo conditions [12]. It is suggested that nitrate serves as an alternative hydrogen sink to lower CH_4_ production in anaerobic ecosystems, but there may be other mechanisms involved as well. However, nitrate metabolites, such as nitrite and nitrous oxide, may suppress methanogenesis directly [1]. To the best of our knowledge, no study is available on the effect of methionine in the presence of nitrate on amino acid metabolism under in vitro conditions.

We hypothesized that the supplementation of methionine in the presence of nitrate in a low-protein diet could increase ammonia incorporation into MCP in the rumen, which might be nutritionally beneficial. Moreover, being a sulfur-containing amino acid, methionine can facilitate balancing sulfur-to-nitrate ratios to maintain the activity of sulfur-reducing bacteria that might enhance nitrate metabolism (by reducing nitrite to ammonia) in the rumen [13]. Therefore, we tested the effect of methionine supplemented in a low-protein diet (roughage 90%, concentrate 10%) on hydrogen balance, cumulative gas, CH_4_ production, rumen fermentation parameters, and microbial populations in the presence of nitrate.

## 2. Materials and Methods

### 2.1. Substrates

We used a low-protein diet consisting of 90% elephant grass and 10% concentrate (finely ground to pass through a 2 mm sieve) as a substrate. Details of the chemical composition of the substrate are given in Table 1.

### 2.2. Treatments

We used sodium nitrate (60 mg/bottle; >99% purity; Baishi Chemical Reagent Co., Tianjin, China) as a basal treatment in all bottles except blank and control. In addition to sodium nitrate, we used three levels of L-methionine (M0, 0%; M1, 0.28%; M2, 1.12%) (≥98% purity; Sigma-Aldrich, St. Loius, MO, USA) to reveal the synergistic effect of NaNO3 on methane production and amino acid metabolism (Table 2).

The control group only consisted of substrate without any treatment. Each treatment group had five bottles as replicates.

### 2.3. In Vitro Batch Culture

The rumen fluid (500 mL) was collected from two rumen-cannulated buffaloes before morning feeding. These buffaloes were fed on the same ration consisting of elephant grass and concentrate *ad libitum*, which was used as substrate for in vitro culture. The collected rumen fluid from the buffaloes was mixed in a single container and strained through two layers of cheese cloth under continuous mixing and N_2_ flushing. Rumen fluid (20 mL) and buffer solution (40 mL) were added to each incubation bottle (containing 0.5 g of substrate), which had each been preheated and flushed with N_2_ [14]. All bottles were sealed, placed in a preheated water bath, and incubated at 39 °C for 72 h with continuous oscillation. Two experimental runs were performed for two consecutive weeks using the same experimental conditions.

### 2.4. Determination of Total Gas and Methane Production

Total gas production was measured in each fermentation bottle at 3, 6, 9, 12, 24, 48, and 72 h of incubation, with a 100 mL lubricated glass syringe attached to the needle, as described previously [15]. The net gas production of a culture bottle (mL) was equal to the gas production in the time period (mL) minus the blank gas production in the corresponding time period (mL). The total cumulative gas production over 72 h was the sum of the net gas production of culture bottles at each time period. At the same time as the gas production measurement at each time point, the CH_4_ content was determined by gas chromatography (GC) (Agilent 7890A, Agilent Technology Company, CA, USA). A sample of 10 μL gas was collected from the fermentation bottle by manual injection needle and was injected directly into GC fitted with an hp-innowax (19091N-133) capillary column measuring 30 m × 0.25 mm × 0.25 μm. The cumulative CH_4_ production over 72 h was calculated as the sum of the actual CH_4_ production at each time period [16].

### 2.5. Determination of Reducing Equivalents (Expressed as H_2_)

The quantity of reducing equivalents (expressed as mmol H_2_ per mL of culture fluid) was calculated as the following sum: 2 equiv. acetate + 1 equiv. propionate + 4 equiv. butyrate + 2 equiv. valerate + 2 equiv. isovalerate. Similarly, the amount of reducing equivalents (expressed as mmol H_2_ per mL of culture fluid) consumed was calculated as the following sum: 2 equiv. propionate + 2 equiv. butyrate + 1 equiv. valerate + 4 equiv. CH_4,_ as described previously [17,18].

### 2.6. Sampling and Determination of In Vitro Fermentation Parameters

At the end of 72 h of in vitro incubation, the fermentation bottles were taken out and put into a mixture of ice and water for 15 min to stop fermentation. Then, each bottle was opened, and the pH was immediately measured with a pH meter (Hanna HI 8424, Shanghai He Yi Instrument Co., Ltd., China). For the determination of ammonia nitrogen (NH_3_-N), 4 mL of the culture filtrate was mixed with 4 mL of 0.2 mol HCl and stored at −20 °C until further analysis. Later, the NH_3_-N content was measured using the indophenols method through a UV-Vis spectrophotometer (PE lambda 35, Shanghai Pudi Biotechnology Co., Ltd. China) at 560 nm wavelength [19]. The microbial protein (MCP) concentration was determined by the Coomassie brilliant blue G250 staining method. The VFA contents were determined by mixing 0.75 mL culture filtrate with an equal volume of 8.2% metaphosphoric acid, and then centrifuging the mixture at 20,000× *g* (4 °C) for 10 min. After centrifugation, 920 µL of supernatant was added to 80 µL internal standard crotonic acid (1 mol/L). Different VFA fractions (C2, C3, C4, C5, iC4, and iC5) were measured using the GC system as described previously [20,21].

### 2.7. Determination of Amino Acid Concentration

Concentrations of individual amino acids were determined through liquid chromatography-tandem mass spectrometry (LC-MS/MS) analysis using a SCIEX Triple Quad 5500 LC-MS/MS System (AB SCIEX (Pvt.) Ltd., Framingham, USA), as reported previously [22]. The LC was conducted with an Acquity UPLC BEH Amide column (1.7 µm, 2.1 mm × 100 mm) (Shanghai Minxin Biotechnology Co., Ltd., Shanghai, China) at 35 °C, with a flow rate of 0.30 mL/min. Mobile phase A consisted of 0.2% formic acid in water (10 mM ammonium formate, 0.2% formic acid) and mobile phase B was 0.2% formic acid in acetonitrile (85/15, 10 mM ammonium formate, 0.2% formic acid). For MS/MS, the parameters included the following: electrospray ionization source set in positive mode, spray voltage at 4500 V, GS1 (atomization gas) at 55 psi, GS2 (auxiliary gas) at 55 psi, scan mode in multiple reaction monitoring, collisionally activated dissociation at medium (collision gas), and atomization temperature at 550 °C.

### 2.8. DNA Extraction and Quantification of Microbial Populations

DNA from the rumen filtrate was extracted using bead beating through the CTAB method, as described previously [23]. The quality and concentration of DNA were determined by a Nanodrop spectrophotometer (Nanodrop ND-2000, Beijing Xinxing qiangsen Biotechnology Co., Ltd. Beijing, China). Quantitative real-time PCR (qRT-PCR) was used to quantify the microbial populations in the rumen fluid by using 16-S (total bacteria and methanogens) and 18-S (fungi and protozoa) primers, as described in our previous study [24]. The primers used for RT-PCR are presented in Table S1. PCR was performed using the SYBRGreen fluorescent dye in a Roche light cycler 480 RT-PCR machine (Roche, Basel, Switzerland). A 20 µL reaction volume containing 9.2 µL SYBR green mixture, 1 µL each of forward and reverse primers of respective species (10 µM), and 8.0 µL nuclease-free water was used for RT-PCR. The amplification profile of RT-PCR for all primer pairs consisted of an initial denaturation for 10 min followed by 40 cycles of 95 °C for 15 s and annealing at 60 °C for 60 s. Standard curves were generated using tenfold serial dilutions of DNA from a pure culture of each microbial species after amplification through conventional PCR (95 °C for 10 s and 60 °C for 60 s, for 40 cycles). The concentration of PCR products were determined by the Nanodrop2000 spectrophotometer. The copy number of each standard was calculated by using the length of PCR product and its respective DNA concentration. The copy number of each unknown sample was calculated through the association of threshold cycle (CT) values to standard curves.

### 2.9. Metagenomic Biomarker Identification and Functional Prediction

The linear discriminant analysis (LDA) effect size (LEfSe) was used to identify predominant bacterial taxa in each treatment group that can be considered as biomarker taxa. The LEfSe is an algorithm approach that utilizes nonparametric Kruskal–Wallis and Wilcoxon rank-sum tests to identify bacterial taxa with significantly different abundances in each treatment group [25]. It also applies LDA to each differentially abundant genus to assess the effect size of respective taxa. In the present study, bacteria taxa having LDA scores (log 10) > 4 were considered to be significantly different. For the prediction of metagenomic functional analysis, the relative abundance of 16S rRNA data was analyzed using PICRUSt 2, as described previously [26].

### 2.10. Statistical Analysis

For each experimental run, the average of five fermentation bottles was taken, which served as the experimental unit for statistical analysis. Data were analyzed by analysis of variance (ANOVA) using the general linear model in SPSS software (SPSS, 2008). Data were analyzed using completely randomized design, having treatment as a fixed effect and the experimental run as a random effect. Tukey’s test was used to reveal the difference among treatment means. Significance was declared at *p* ≤ 0.05. The abundances of bacterial phyla and genera were compared using the Kruskal–Wallis H test with a false discovery rate (FDR) correction and Scheffer as a post-hoc test to elucidate differences across treatment groups. Spearman’s rank correlation (r) analyses were performed with the vegan R package (version 3.2) to analyze the association of relative abundance of bacterial genera with VFA, total gas, H_2_, CH_4_, and amino acid contents. Correlation heat maps were constructed using the corrplot R package. In the two-dimensional heat map, changes in defined color and its depth indicate the nature and strength of the correlation, respectively. Asterisk signs (*) were used when the r values were >0.4 and the *p* values were <0.05 (* 0.01 < *p* ≤ 0.05, ** 0.001 < *p* ≤ 0.01, *** *p* ≤ 0.001).

## 3. Results

### 3.1. Hydrogen Balance, Total Gas, and CH_4_ Production

**Figure 1:**
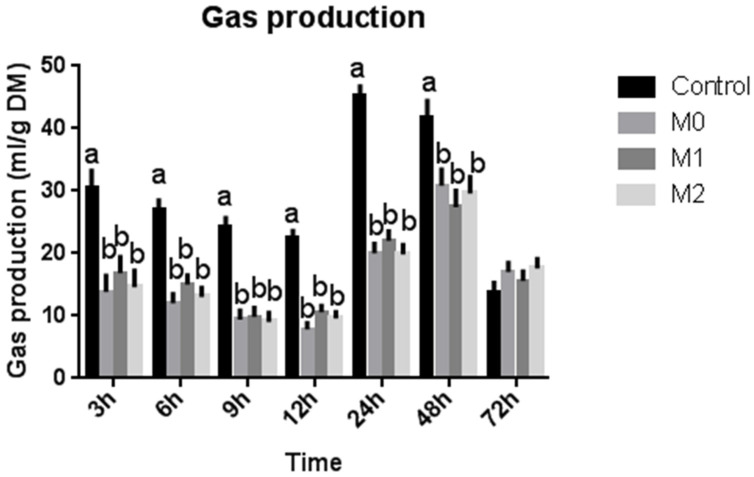
Total gas production at different time intervals (bars at one time interval with different letters indicate significant difference at *p* < 0.05).

**Figure 2:**
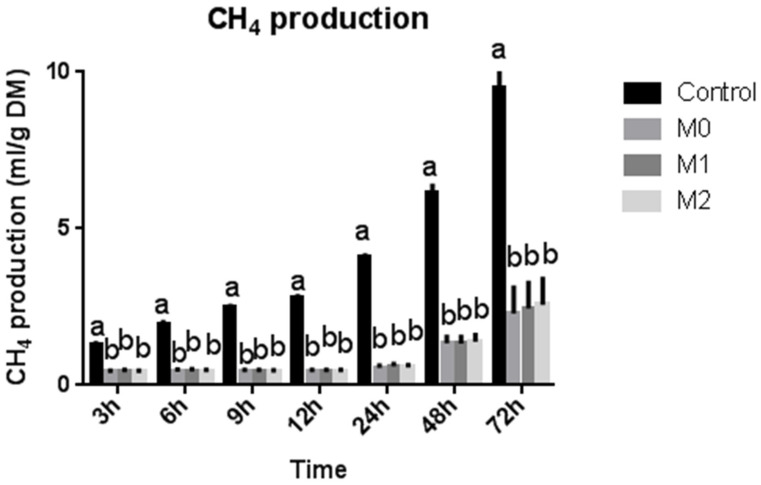
Total methane production at different time intervals (bars at one time interval with different letters indicate significant difference at *p* < 0.05).

Treatment (M0, M1, and M2) decreased (*p* = 0.001) the H_2_ produced and utilized, leading to a reduced H_2_ recovery percentage compared to the control group (Table 3). However, no difference in hydrogen balance was observed within treatment groups. Treatment decreased (*p* = 0.001) the total gas and CH_4_ production compared to the control group, but no difference was observed among different treatment groups (Table 3). The results revealed a steady decrease in gas production from 3 h up to 12 h of incubation; after that, it started increasing up to 48 h. After that, it once again exhibited a decline (Figure 1). Contrarily, CH_4_ yield exhibited an initial steady increase up to 12 h; after that, it showed a sharp continuous increase up to 72 h in the control group (Figure 2). However, the treatment groups (M0, M1, and M2) showed almost no change in CH_4_ yield up to 12 h; after that, it started increasing steadily at a much slower rate than the control group.

### 3.2. Rumen Fermentation Parameters

Treatment affected all fermentation parameters except acetate and NH_3_-N, which did no exhibit any change. Both methionine and nitrate increased (*p* = 0.001) the pH of buffered rumen fluid compared to the control group (Table 4). M0 and M2 showed higher pH values than M1 (6.84 and 6.80 vs. 6.78, respectively), when compared among treatment groups. Methionine exhibited no effect on other rumen fermentation parameters. Treatment with nitrate decreased (*p* = 0.011) the propionate, isobutyrate, butyrate, isovalerate, and valerate compared to the control group. Similarly, treatment also decreased (*p* = 0.001) the TVFA compared to the control. However, treatment increased (*p* = 0.001) the MCP and acetate/propionate ratio (A/P ratio) compared to the control.

### 3.3. Ruminal Amino Acids

The results revealed that treatment significantly altered amino acid metabolism (Table 5). The concentration of total and individual essential amino acids (including histidine, isoleucine, leucine, lysine, methionine, phenylalanine, threonine, tryptophan, and valine) was higher (*p* < 0.05) in M0 and M1 compared to M2 and control group. Similarly, treatment increased the concentration of total and individual non-essential amino acids (including alanine, arginine, glycine, glutamine, glutamate, proline, tyrosine, serine, and aspartic acid) in M0 and M1 compared to M2 and the control group. However, treatment showed no effect (*p* > 0.05) on cysteine, while methionine supplementation (M1 and M2) increased (*p* = 0.003) the asparagine concentration compared to the control and M0 groups.

### 3.4. Rumen Microbial Populations

Treatment increased (*p* = 0.001) the rumen population of total bacteria and methanogens but no effect on total fungal count was observed compared to the control group (Table 6). The highest protozoa count was observed in M1 compared to the control group; however, the other two treatments showed similar protozoa counts.

### 3.5. Rumen Bacterial Diversity

#### 3.5.1. Alpha and Beta Diversity Parameters

Treatment reduced (*p* = 0.001) Shannon’s index while increasing Simpson’ index of alpha diversity of rumen bacteria compared to the control group (Table 7). However, treatment showed no effect on the number of observed species (Sobs) or the ACE and Chao indices. Moreover, treatment decreased (*p* = 0.001) Shannon’s evenness and Simpson’s evenness compared to the control group.

**Figure 3:**
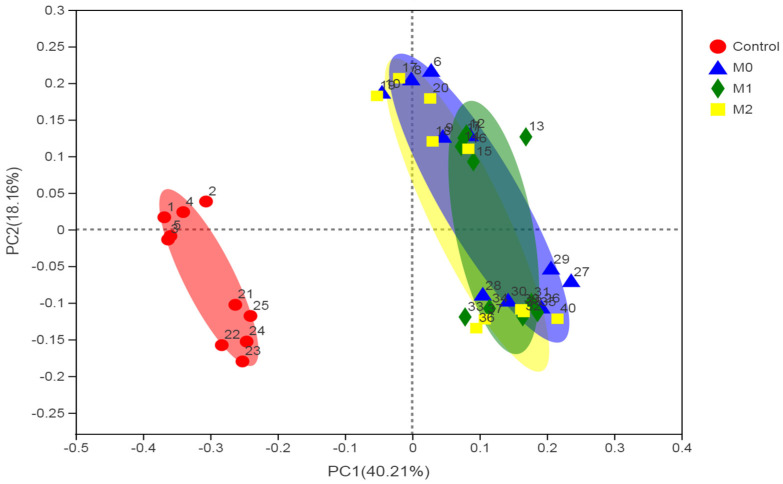
First two dimensions from the (non-metric) multi-dimensional scaling of the Bray-Curtis dissimilarity matrix. Samples were grouped by treatment. PERMANOVA amongst all groups *p* = 0.001 (using 999 permutations).

Beta diversity was determined through (non-metric) multi-dimensional scaling (NDMS) of the Bray-Curtis dissimilarity matrix using PERMANOVA with 9999 permutations, which showed a significant effect (*p* = 0.001) from treatment (Figure 3).

#### 3.5.2. OTU Statistics

**Figure 4:**
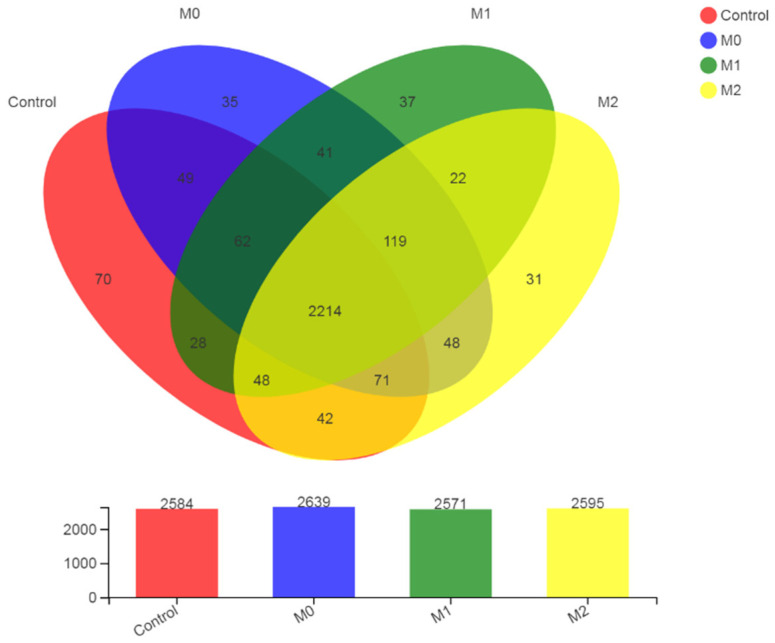
OUT distribution across different treatment groups.

Results revealed a total of 2971 OTUs belonging to 729 species, 381 genera, 187 families, 112 orders, 44 classes, and 21 phyla. The highest number of OTUs was found in M0, followed by M2, control, and M1 (Figure 4). A greater number of OTUs (2214) was shared among the four groups. The highest number of unique OTUs (71) was observed in the control group, followed by M1 (37), M0 (35) and M2 (31).

#### 3.5.3. Relative Abundance of Bacterial Phyla

**Figure 5:**
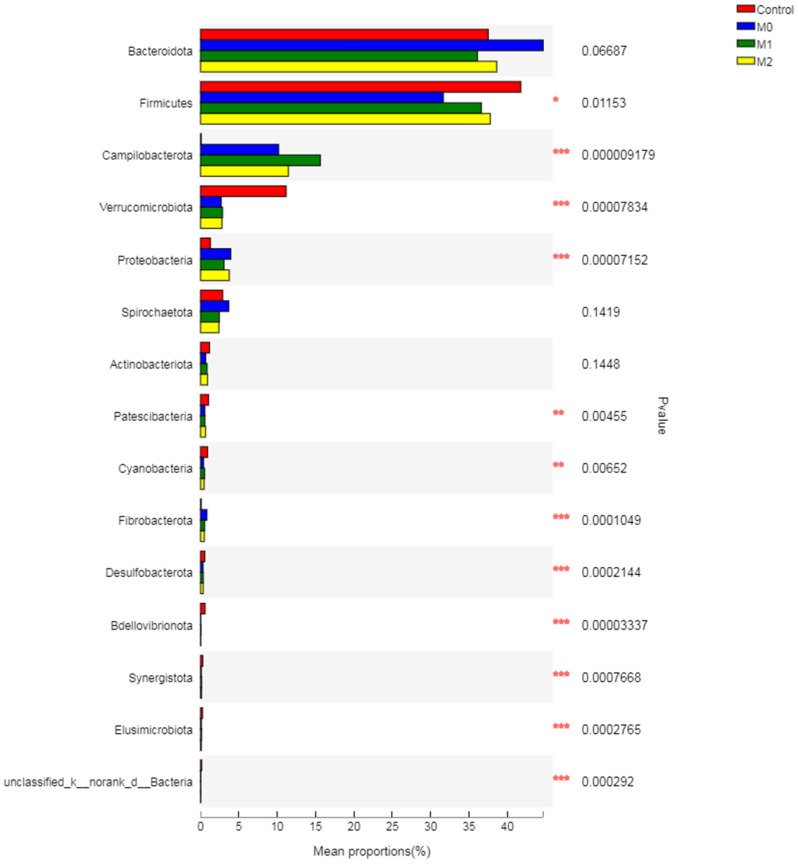
Relative abundance of different bacteria phyla across different treatment groups (Kruskal–Wallis H-Test with *p* < 0.05 as a significant difference).

Treatment showed no effect (*p* = 0.1) on the relative abundance of Bacteroidetes but decreased (*p* = 0.019) the Firmicutes, compared to the control group (Figure 5; Table S2). Methionine seemed to alleviate the adverse effect of nitrate on the relative abundance of Firmicutes by revealing higher abundances in M1 (36.62%) and M2 (37.80%) compared to M0 (31.67%). Treatment also increased (*p* = 0.001) the relative abundance of Campilobacterota and Proteobacteria compared to the control. However, treatment substantially decreased (*p* = 0.001) the Verrucomicrobiota in the rumen. Treatment also exhibited negative effects on Patescibacteria and Cyanobacteria by decreasing (*p* < 0.05) their population, compared to the control group.

#### 3.5.4. Relative Abundance of Bacterial Genera

**Figure 6:**
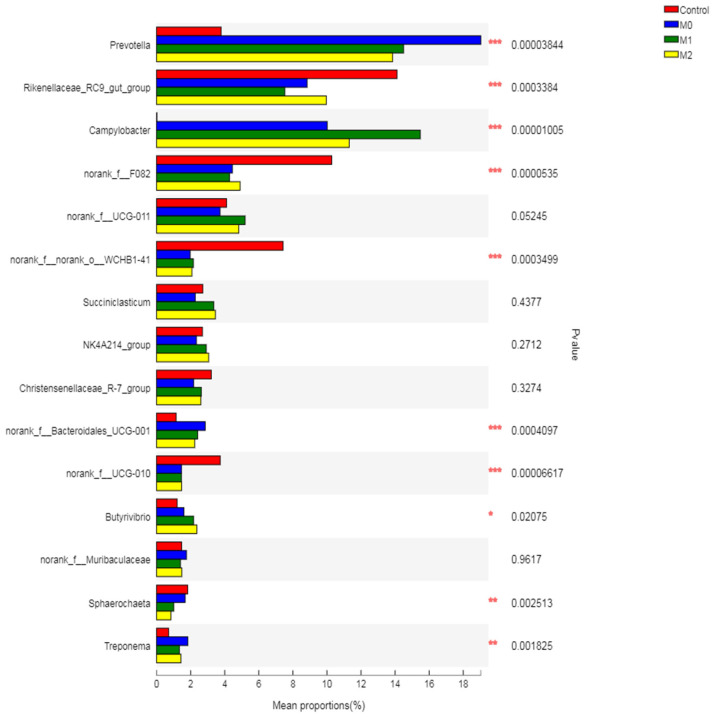
Relative abundance of different bacteria genera across different treatment groups (Kruskal–Wallis H-Test with *p* < 0.05 as a significant difference).

Treatment increased (*p* = 0.001) the *Prevotella* compared to the control; however, a higher relative abundance of this genus was observed in the M0 group (19.03%) compared to the M1 (14.5%) and M2 (13.85%) groups (Table S3). In contrast, treatment decreased (*p* = 0.001) the abundance of *Rikenellaceae_RC9_gut_group* compared to the control group (Figure 6). Methionine showed more pronounced effects on the relative abundance of *Campylobacter*, which was substantially increased in M1 (15.48%) compared to M0 (10.02%), M2 (11.31%), and the control group (0.02%). Treatment showed no effect on the relative abundances of *norank_f__UCG-011, Succiniclasticum, NK4A214_group, Christensenellaceae_R-7_group, norank_f__Muribaculaceae, Saccharofermentans, Ruminococcus, Lachnospiraceae_NK3A20_group* and *Prevotellaceae_UCG-003*. Treatment decreased (*p* < 0.01) the population of *norank_f_F082, norank_f__norank_o__WCHB1-41, norank_f__UCG-010* and *Sphaerochaeta* compared to the control. However, treatment increased (*p* < 0.05) the relative abundance of *Treponema* and *norank_f__Bacteroidales_RF16_group* compared to the control group.

### 3.6. Biomarker Bacteria Taxa and Metagenomic Functional Profile

**Figure 7:**
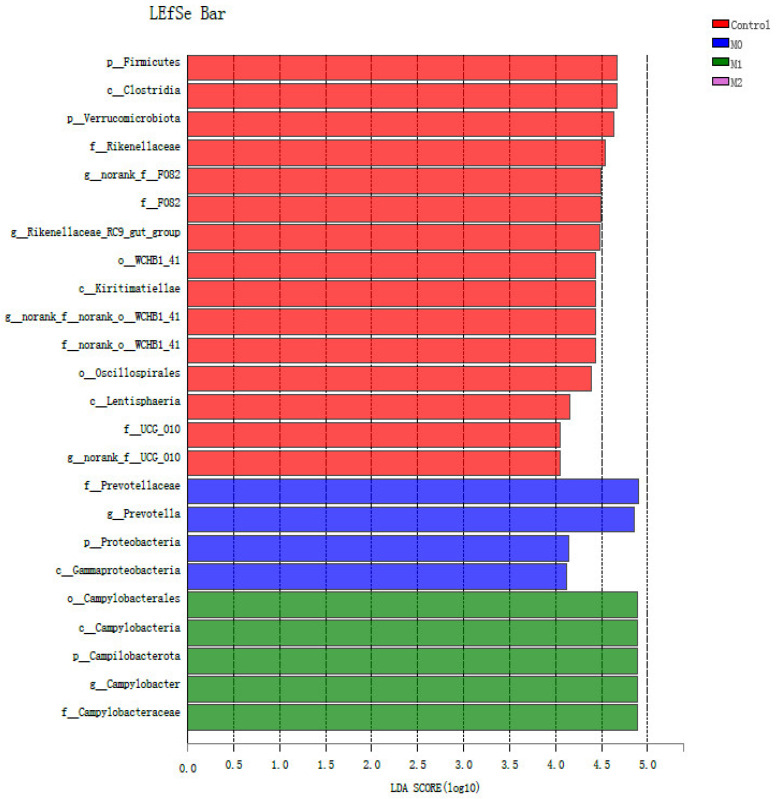
Biomarker bacterial genera in different treatment groups as revealed by LEfSe (LDA)-based analysis (LDA > 0.4).

**Figure 8:**
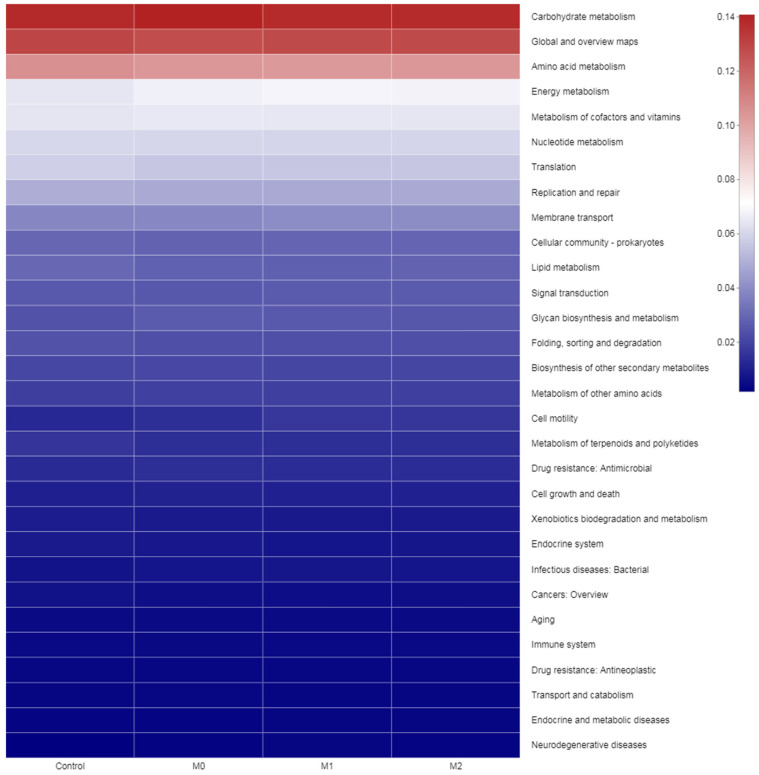
Top KEGG enriched pathways in different treatment groups.

We identified bacterial taxa that were predominantly abundant as biomarkers among the treatment groups through LEfSe. A total of 24 significant taxonomic clades (LDA score > 4) were identified with six genera biomarkers (Figure 7). The highly selected bacterial genus in the methioninegroup was *Campylobactor*. However, two bacterial taxa (*Prevotella* and *γ-Proteobacteria*) were identified as biomarkers in the nitrate group. Four genera, namely *F-082, Rikenellaceae_RC9_gut_group, norank_f__norank_o__WCHB1-41* and *norank_f__UCG-010*, were highly affected in the control group. Metagenomic functional prediction revealed 30 enriched KEGG pathways (with >1% relative abundance), as shown in Figure 8. The three most abundant pathways included carbohydrate metabolism (with an abundance of 13.9, 14.1, 13.8, and 13.8% in control, M0, M1, and M2, respectively), amino acid metabolism (with an abundance of 10.6, 10.4, 10.3, and 10.4% in control, M0, M1, and M2, respectively), and energy metabolism (with an abundance of 6.4, 6.7, 6.8, and 6.8% in control, M0, M1, and M2, respectively).

### 3.7. Association of Rumen Bacteria with Ruminal Gas, VFA, and Amino Acid Contents

**Figure 9:**
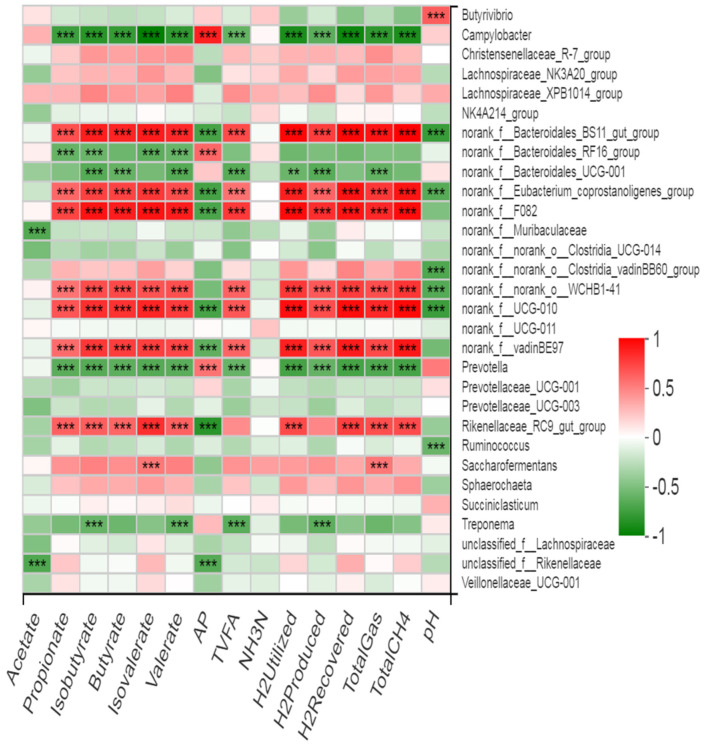
Correlation of bacterial genera with hydrogen balance, total gas, CH_4_, and VFAs.

Our findings revealed that six bacterial genera showed positive correlation (*p* < 0.001, r > 0.5) with gas, CH_4_, ruminal hydrogen balance (H_2_ produced, utilized, and recovery), and VFAs including propionate, butyrate, isobutyrate, valerate, and isovalerate (Figure 9). These genera showed no significant correlation with acetate content but were negatively correlated (*p* < 0.001, r > 0.5) with the acetate-to-propionate ratio and ruminal pH. However, two bacterial genera (*Complylobacter* and *Prevotella*) exhibited negative correlation (*p* < 0.001, r > 0.5) with gas, CH_4_, ruminal hydrogen balance (H_2_ produced, utilized, and recovery), and VFAs (except the A/P ratio). *Treponema* also showed negative correlation (*p* < 0.001, r > 0.5) with H_2_ produced, TVFAs, isobtyrate, and valerate. Two uncharacterized genera (*UCG-01* and *RF-16 group*) of Bacteroidales also showed negative correlation with ruminal gas and VFA contents. *Saccharofermentans* showed positive correlation with total gas and isobutyrate (*p* < 0.001, r > 0.5). Two uncharacterized genera (one each from the families Muribaculaceae and Rikenellaceae) showed negative correlation with acetate content. *Butyrivibrio* showed a positive correlation while *Ruminococcus* and *Clostridia_vadinBB60_group* showed a negative correlation with ruminal pH.

**Figure 10:**
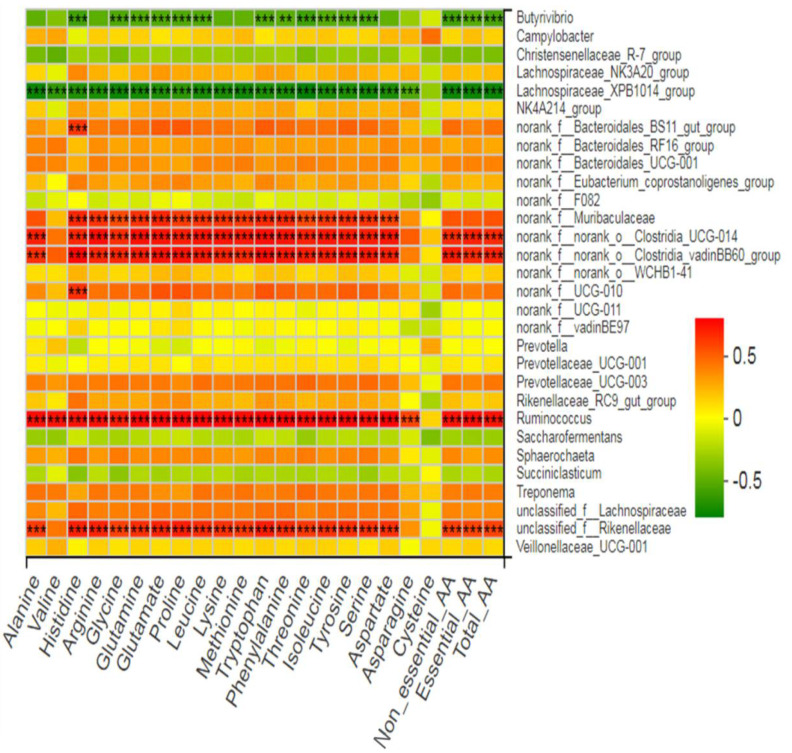
Correlation of bacterial genera with ruminal amino acid contents.

Five bacteria genera (*Clostridia_UCG-014, Clostridia_vadinBB60_group, Ruminococcus, norank_f__ Muribaculaceae,* and *unclassified_f__Rikenellaceae*) showed a positive correlation (*p* < 0.001, r > 0.5) with individual AA (except valine and cysteine) and total essential and non-essential AA contents (Figure 10). *Lachnospiraceae_XPB1014_group* showed a negative correlation (*p* < 0.001, r > 0.5) with individual AA (except cysteine) and total essential and non-essential AA contents. Similarly, *Butyrivibrio* also showed a negative correlation (*p* < 0.001, r > 0.5) with individual AA contents (except aspartate, asparagine, cysteine, lysine, methionine, arginine, valine, and alanine) and total essential and non-essential AA contents. Two uncharacterized genera (*Bacteroidales_BS11_gut_group* and *f__UCG-010*) only showed a positive correlation with histidine.

## 4. Discussion

### 4.1. Effect of Treatment on H_2_ Balance, Cumulative Gas, and CH_4_ Production

It has been well established that nitrate supplementation in ruminants can decrease their methane emissions. This is mainly attributed to the fact that nitrate can serve as an alternative hydrogen sink in anaerobic ecosystems and drives metabolic hydrogen away from methanogenesis. However, other mechanisms may also be involved, but nitrate metabolites such as nitrite and nitrous oxide have been shown to directly suppress the process of ruminal methanogenesis [1]. In addition to hydrogen and CO_2_, methanogens also use other substrates such as methyalmines to produce CH_4_, as studies have revealed that methylotrophic methanogens (*Thermoplasmata*) can metabolize methylamines [27]. Taking this all into account, we endeavored to determine whether methionine can affect methanogenesis through methylamines in the presence of dietary nitrate in a low-protein diet.

Our findings revealed that methionine did not affect the total gas and CH_4_ production in the presence of nitrate at each time point from 0 to 72 h of incubation. Overall, supplementation of nitrate resulted in a 25% decrease in CH_4_ production compared to the control group, but no difference was observed among different treatment groups. Similarly, total gas production decreased by up to 204% in response to treatment compared to the control group, while revealing no difference within the treatment groups. Our findings agree with earlier studies reporting negative effects of nitrate on in vitro methanogenesis [11,28,29,30,31,32,33,34,35].

Similar findings have also been reported by in vivo studies demonstrating beneficial effects of nitrate supplementation on CH_4_ emission. Recently, a study showed that long-term supplementation of encapsulated nitrate persistently decreased enteric CH_4_ emissions in grazing steers, mainly by decreasing the relative abundance of archaea (Methanobrevibacter) in the rumen [12].

The major anti-methanogenic effect of nitrate is attributed to the reduction of nitrate by rumen microbes into nitrite and subsequently into NH_3_ (through nitrate and nitrite reductases), which can thermodynamically outcompete methanogenesis [36,37]. The beneficial effects of CH_4_ inhibition by nitrate stems from not only directing metabolic H_2_ away from methanogenesis, but also reducing the relative abundance of H_2_-producing bacteria (mainly Firmicutes) [38]. Similar findings were observed in the present study, as nitrate significantly decreased the H_2_ produced and utilized, while also decreasing the H_2_ recovery percentage and significantly decreasing the relative abundance of Firmicutes. Moreover, nitrate supplementation has been shown to stimulate the growth of NC10 bacteria that oxidize methane, leading to lower overall CH_4_ production [31]. Interestingly, methionine alleviated the adverse effects of nitrate on Firmicutes by increasing their relative abundance (6–7%) compared to the M0 group, despite no increase in CH_4_ production. This implies that methionine can support diet degradability in the presence of nitrate, as Firmicutes are a major carbohydrate-fermenting bacteria in the rumen [39]. However, further studies are required to corroborate these findings, as we did not determine the diet degradability in the present study.

### 4.2. Rumen Fermentation Parameters

Dietary supplementation of nitrate usually results in higher ruminal NH_3_ concentrations, mainly because reduction of nitrate leads to nitrite production, which competes with CO_2_ for H_2_ [40,41]. However, in the present study, we did not observe any change in NH_3_ concentration, in spite of a significant decrease in CH_4_ production, which means that spared H_2_ was utilized by pathways other than NH_3_ formation. Also, no effect of nitrate supplementation on NH_3_ concentration under in vitro conditions had been reported earlier [5,42]. However, some studies have also reported a negative effect of nitrate supplementation on NH_3_ concentration [43], which reveals that conversion to NH_3_ is not solely responsible for nitrate metabolism in the rumen [13]. No effect of methionine was observed on individual or total VFAs, which agrees with earlier studies on in vitro continuous cultures of rumen microbes [44]. Moreover, supplementation of methionine in in vitro and in vivo studies in cattle showed no effect on rumen fermentation and N digestion [45].

Methane inhibition is generally expected to direct rumen fermentation from acetate towards propionate production [46], which has been extensively reported in batch culture studies, as revealed by a meta-analysis [1]. In the present study, we did not observe any change in acetate concentration in response to treatment, but a significant increase in the acetate-to-propionate ratio was observed, which is in agreement with earlier reports [7,47]. This is mainly attributed to the higher release of H_2_ and enhanced growth and activity of acetate-producing microbes [40]. It has been well established that ruminal acetate is produced as a result of the ruminal fermentation of cellulose and hemicellulose [48], accompanied by H_2_ production, which is utilized for nitrate reduction in the rumen. This utilization of H_2_ alleviates the potential adverse effect of H_2_ on the rumen fermentation process, particularly fiber degradation and acetate production [41,49]. This may be the reason why we did not observe any decrease in acetate, as found in all other VFAs in the present study. However, treatment increased the A/P ratio compared to the control, which is in agreement with earlier studies [15,29].

Treatment increased (*p* = 0.001) the pH of buffered rumen fluid compared to the control group. The M0 and M2 groups showed higher pH than the M1 group (6.84 and 6.80 vs. 6.78, respectively). Similar findings had been reported earlier by [6], regarding the significant increase in pH of ruminal fluid in response to nitrate supplementation in in vitro batch cultures.

In the present study, nitrate supplementation decreased propionate, isobutyrate, butyrate, isovalerate, and valerate compared to the control group. Consequently, the concentration of TVFA also decreased in the supplemented groups. These findings are in agreement with earlier in vitro studies involving nitrate supplementation [10,15,29,31]. Moreover, the decrease observed for isobutyrate and isovalerate in the present study is also in line with earlier studies that reported CH_4_ inhibition with nitrate supplementation in batch cultures [15,33,35]. These findings are mainly attributed to the toxic effects of nitrate on rumen bacteria and its strong inhibitory action on the in vitro rumen fermentation process [42,50].

The significant increase in MCP observed in response to treatment in the present study is favorable, particularly in the case of a low-protein diet. These findings are in agreement with earlier studies that reported an increase in MCP under in vitro [10,17] and in vivo [51] conditions. Many studies have suggested that nitrate supplementation could serve as a non-protein nitrogen source (NPN) under certain dietary conditions (such as low-protein diets) and can even effectively replace other NPN sources (such as urea) to facilitate synthesis of MCP in the rumen [52,53,54,55,56]. Efficiency of rumen fermentation largely depends on the growth of microbes, which requires an abundant and continuous source of energy [57]. Reductive processes involved in energy conservation for growth are ubiquitously advantageous for microbes which perform these processes. Thermodynamically, the reduction of nitrate is more beneficial than methanogenesis from an energy conservation point of view, and therefore, might facilitate higher microbial growth [37]. Under in vivo conditions, nitrate supplementation has also been shown to redirect metabolic H_2_ away from CH_4_ formation, subsequently resulting in enhanced incorporation of NH_3_ into MCP in the rumen of dairy cows [58].

Earlier studies have demonstrated that nitrate acts as N source in the rumen and could increase microbial cell synthesis by increasing ATP yield through dissimilatory reduction [59]. Nitrate has been shown to serve as an electron acceptor for *Clostridium perfringens*, leading to enhanced growth rates [60], and the stimulatory effect of nitrate on microbial nitrogen synthesis is consistent with the thermodynamically relevant electron tower concept [61]. We observed no effect of methionine on rumen fermentation parameters and microbial growth, which is consistent with earlier studies reporting no effect of amino acid (tryptophan) supplementation on microbial fermentation or growth rate under in vitro conditions [10].

### 4.3. Ruminal Amino Acids

Dietary amino acids generally have a stimulatory effect on the growth of rumen microbes, even in the case of abundant NH_3_ and carbohydrates [62,63]. Even though rumen bacteria do not have AA requirements per se, they do respond to AA supplementation with improved growth efficiency [63,64]. Supplementation of AA has shown to stimulate fibrolytic bacteria [65]. Moreover, growing *Streptococcus bovis* in a medium containing AA resulted in low wastage of energy as heat, suggesting an increased efficiency of energy and carbon use in the presence of AA [66]. Supplementation of a mixture of 20 AA resulted in increases in growth rate by 46% and efficiency by 15% [67]. Similarly, many other in vitro studies revealed that growth of cellulolytic bacteria is stimulated by dietary peptides or amino acids [68,69]. However, it is hypothesized that the inhibition of methanogenesis leads to inhibiting amino acid fermentation, resulting in decreased deamination of amino acids. However, the amount of metabolic hydrogen produced or utilized during the fermentation of protein is difficult to determine, mainly due to the involvement of several pathways in hydrogen utilization leading to the production of each amino acid [18]. In the present study, we endeavored to evaluate the effect of methionine supplementation on amino acid metabolism in the presence of nitrate under in vitro conditions of rumen fermentation.

The present study revealed that a lower level of methionine exhibited no effect on AA metabolism in the presence of nitrate. Our findings indicated that nitrate alone (M0) and in combination with a low level of methionine (0.28%) significantly increased the concentration of total and individual essential and non-essential AA, compared to the higher level of methionine and control groups. However, the higher level of methionine (1.12%) exhibited a negative effect on ruminal AA contents (except cysteine and asparagine). These findings are in agreement with earlier studies reporting adverse effects of higher levels (>2 mmol/L) of AA (valine, leucine, or isoleucine) on fiber degradation in a wheat straw substrate under in vitro conditions [70]. These findings imply that rumen microbes require optimal concentrations of the AA as higher levels do not improve fiber degradation and even exert negative effects on rumen fermentation.

However, in the present study, methionine supplementation (M1 and M2) increased (*p* = 0.003) the asparagine concentration compared to the control and M0 groups. No effect of methionine on cysteine content and a positive effect on asparagine content reveals that the metabolic pathways are different for these AA, owing to the involvement of different microbial communities. In vitro studies on AA metabolism under the inhibition of methanogenesis have revealed that nitrate increase the aspartate content [6]. Contrary to earlier reports, we observed a significant increase in the ruminal AA contents in response to nitrate treatment, which indicated redirection of metabolic H_2_ into the synthesis of microbial N and protein. These findings are in line with the higher MCP contents observed in the present study. There are limited studies reporting changes in AA content in response to nitrate treatment; however, [6] reported that inhibiting methanogenesis through nitrate can yield inconsistent results.

### 4.4. Rumen Microbial Populations

Undoubtedly, nitrate significantly decreased CH_4_ production in the present study, but at the same time, the population of total bacteria and methanogens was also increased. Similar findings have been reported earlier in steers fed with different levels of nitrate, revealing no change in the population of total methanogens but an observed increase in the relative abundance of *Methanosphaera* and *Methanimicrococcus* [31]. However, studies have also reported a decrease in populations of methanogens in response to nitrate treatment under in vitro conditions [40]. These contrasting findings may be attributed to the type of substrate and, most importantly, the level of nitrate, as it is toxic to rumen microbes at higher concentrations, whilst low nitrate levels have been shown to increase the relative abundance of rumen bacteria [33,35]. Studies have reported that 1% nitrate promoted the growth of rumen bacteria, but 2% nitrate showed strong inhibitory action against them [49]. This might be one of the reasons that we observed an increase in total methanogens and bacteria in response to nitrate supplementation (at 1% inclusion rate), which is in agreement with earlier studies [71]. Moreover, studies have shown that microbial communities can adapt to dietary nitrate by increasing the population of nitrate-reducing bacteria [59], which may be another possible reason why a substantial increase in the relative abundance of the *Campylobacter* genus was observed in all treatment groups.

However, studies have reported that cellulolytic bacteria (*F. succinogenes*) are inhibited by nitrate inclusion in the nitrate under un-adapted rumen under in vitro culture [7,35]. The adverse effect of nitrate on the ruminal ecosystem is mainly associated with inhibition of the electron transport system of microbes manifested by high nitrite levels [35,72]. This is why microbes that do not possess an electron transport system are less likely to be affected by nitrate supplementation. Furthermore, lower levels of nitrate are safe for dietary supplementation, as long as the nitrite levels do not exceed the capacity of nitrite-reducing bacteria [73].

The decrease in CH_4_ production in the presence of an increase in total methanogens reveals that decreasing the population of methanogens might not necessarily lead to mitigating CH_4_ emission, and vice versa, at least within a short period of time. A decrease in the population of methanogens with no corresponding decrease in CH_4_ production [15] reveals that additives like dietary nitrate can affect methanogens and CH_4_ production in different ways. Moreover, methionine showed a significant increase in the total protozoa content, which is in agreement with earlier studies that reported an increase in protozoa count in response to methionine or its analogue under in vitro and in vivo conditions [74,75]. Patton et al. [74] suggested that methionine caused an increase in the protozoa biomass, owing to the fact that it serves as a methyl donor to produce phosphatidylcholine. Since free choline is rapidly degraded in the rumen [72], protozoa are the primary suppliers of phosphatidylcholine, an important molecule used in the packaging of fatty acids into very low-density lipoproteins and chylomicrons [76].

Ruminal protozoa play crucial role in dietary fermentation as well as interacting with other rumen microbial communities; consequently, they affect the quantity and proportion of CH_4_ and other end products of rumen fermentation [77,78]. Protozoa are capable of engulfing organic matter (soluble proteins) and bacteria, and also perform hydrolysis and fermentation of ingested material. The major VFAs produced by protozoa are acetate and butyrate [77,79]. This indicates that protozoa play a crucial role in rumen fermentation. The increase observed in protozoa count may be responsible for the higher acetate-to-propionate ratio reflected in response to methionine supplementation in the present study.

### 4.5. Ruminal Bacterial Diversity

Studies have shown that nitrate supplementation in ruminants can shift the composition of rumen bacterial communities [30] through nitrite toxicity (a nitrate reduction pathway intermediate), by creating competition for hydrogen and changing the ruminal pH [10,35]. Ruminants’ diets contain fiber as a major ingredient, which is fermented by cellulolytic bacteria to yield VFA, which are essential for rumen microbes and also serve as an energy source for the host. Due to the inhibitory action of nitrate on methanogenesis, it is presumed that both nitrate- and nitrite-reducing microbes compete with methanogens for H_2_ in the rumen [59,80,81]. Nitrate is toxic to rumen bacteria at higher concentrations (>12 mmol/L), but at optimum levels (5 mmol/L), it can increase the relative abundance of cellulolytic bacteria [33,35]. Some studies have also reported no effect of nitrate on total bacterial population under in vitro ruminal cultures [15].

The present study showed that both nitrate and methionine showed no significant effect on the relative abundance of total Bacteroidetes, which is in agreement with earlier studies [51]. But at the genus level, treatment significantly increased the *Prevotella* compared to the control group, revealing a higher abundance of this genus in the M0 group (19.03%) compared to M1 (14.5%) and M2 (13.85%). These findings agree with earlier studies reporting a significant increase in the relative abundance of *Prevotella* with 1% nitrate supplementation [49]. However, methionine showed a relatively negative effect on *Prevotella* compared to nitrate supplementation alone. This is mainly attributed to the fact that *Prevotella* is one of the major H_2_-utilizing bacteria, so an increase in its abundance in response to a higher availability of H_2_ is expected under CH_4_-inhibiting conditions, as reported previously [82].

Low and high levels of methionine exhibited a 1.2- and 0.9-fold decrease in the relative abundance of Bacteroidetes, respectively, with a corresponding 0.9- and 1.2-fold increase in the Firmicutes. This may be due to a negative association of Bacteroidetes with Firmicutes in the ruminal ecosystem. The present study revealed a negative effect of nitrate on Firmicutes, but methionine alleviated the toxic effect of nitrate by enhancing their abundance. After *Prevotella*, the major genera in Bacteroidetes were *norank_f_F082* and *Rikenellaceae_RC9_gut_group*, which were substantially decreased by the treatment. However, *norank_f__Bacteroidales_RF16_group* of this phylum was increased by methionine compared to the control and nitrate groups, revealing a positive effect of methionine on this genus.

However, both nitrate and methionine significantly increased the relative abundance of Campilobacterota and Proteobacteria in the present study, which is in agreement with earlier studies [49]. Nitrate substantially increased (500-fold) the relative abundance of *Campylobacter* compared to the control group. Interestingly, combination of methionine and nitrate also exhibited a stimulatory effect on *Campylobacter*, as revealed by a 1.5- and 1.2-fold increase in M1 and M2, respectively, compared to the M0 group. *Campylobacter* is a Gram-negative non-fermentative bacterium, and its many species (such as *C. jejuni*, *C. lari*, *C. concisus*, *C. fetus*, and *C. coli* etc.) possess the nitrate reductase gene [83,84]. Studies have shown that the inclusion of nitrate significantly increases the abundance of nitrogen-reducing species of *Campylobacter* (C. fetus) in the rumen [49]. Our findings are in line with this study, as they reported a significant increase in nitrate-reducing bacteria in response to nitrate supplementation. The positive effect of methionine at a lower dose on the *Campylobacter* genus reveals that it increased the relative abundance of sulfur-reducing species of this genus. Moreover, the increase in *Campylobacter* in response to methionine is advantageous for balancing the sulfur-to-nitrate ratio, which is a primary condition for optimum utilization of nitrate to avoid nitrite accumulation and subsequent toxic effects on the rumen ecosystem [13].

In the present study, a substantial decrease in the phylum Verrucomicrobiota in the rumen in response to nitrate treatment was also observed, which is in agreement with earlier reports [49]. The major effect of nitrate was observed on the major genus of this phylum, which is an uncharacterized bacteria named as *f__norank_o__WCHB1-41*. Treatment also exhibited negative effects on Patescibacteria and Cyanobacteria by decreasing their population compared to the control group. Cyanobacteria are common rumen bacteria with a crucial role in nitrate assimilation and reduction of methanogenesis [55,85]. Studies have shown an increase in the relative abundance of Cyanobacteria in response to low levels of nitrate inclusion under in vitro conditions [49], but we observed a low abundance (<1%) of this phylum in our study.

Methionine showed no substantial effect on Proteobacteria, but nitrate significantly increased this phylum, revealing a positive effect on its population. These findings are in line with earlier studies that reported a negative association of Proteobacteria with CH_4_ emissions [86,87,88]. This is mainly attributed to the fact that some members of the phylum (particularly γ-Proteobacteria) produce succinate as an intermediate of propionate or degrade lactate, resulting in less H_2_ release in comparison to other VFAs, which subsequently decreases the rate of methanogenesis [46]. Additionally, some species of Proteobacteria are also involved in the biosynthesis of branched-chain or aromatic amino acids (the shikimate pathway), which has been considered to be a strong indicator of low CH_4_ emissions in sheep [89]. Therefore, our findings support the idea that γ-Proteobacteria are a promising candidate for devising nutritional interventions to mitigate CH_4_ emissions in ruminants without negatively affecting feed efficiency [90].

### 4.6. Biomarker Bacterial Genera and Functional Prediction Profile

Our findings revealed that nitrate resulted in a significant shift in the relative abundance of *Prevotella* and γ-Proteobacteria. This is mainly because these bacteria utilize H_2_ via the succinate pathway for propionate production through the fermentation of sugars and lactate [91,92]. It is possible that these pathways were upregulated in response to the anti-methanogenic action of nitrate, as these are major routes for H_2_ consumption [93]. These findings agree with earlier studies reporting a positive association of bacterial OTUs (belonging to *Prevotella* spp.) that increased in a dose-dependent fashion in response to anti-methanogenic treatment (BCM) in the rumen [94] The *Campylobacter* genus was identified as a biomarker in the methionine group (low dose). This genus contains both nitrate-reducing and sulfur-reducing bacteria, and their increase in response to a low dose of methionine is possibly associated with the maintenance of the sulfur-to-nitrate ratio in the ruminal culture. This shift in the ruminal ecosystem is important for the optimum utilization of nitrate to avoid nitrite accumulation and subsequent toxic effects on the rumen ecosystem [13].

Metagenomic functional prediction revealed 30 enriched KEGG pathways including carbohydrate, amino acid, and energy metabolism pathways with the highest abundance. No substantial change in the top three pathways in the different treatment groups revealed the functional redundancy of the microbial ecosystem, as these pathways did not significantly differ in spite of substantial changes observed in the relative abundance of rumen bacteria.

### 4.7. Association of Rumen Bacteria with Ruminal Gas, VFA, and Amino Acid Contents

Our findings revealed that fibrolytic bacteria showed a positive association with gas, CH_4_, ruminal hydrogen balance (H_2_ produced, utilized, and recovery), and VFAs (including propionate, butyrate, isobutyrate, valerate, and isovalerate), which agrees with earlier studies [87]. This is mainly attributed to the fact that fibrolytic bacteria (particularly cellulytic bacteria, such as *Ruminococcus*, and several species of Firmicutes, such as *Eubacterium spp*) are well known H_2_ producers. On the other hand, *Fibrobacter* is the well-known cellulolytic bacteria that does not produce H_2_, while Bacteroidetes are mainly responsible for H_2_ utilization [38]. Moreover, it is reported that an abundance of fiber-degrading bacteria explains much of the variability (~50%) in the CH_4_ production in ruminants [90]. Additionally, fiber is the main substrate to produce VFAs in the rumen. That is why fiber degrading bacteria showed a positive correlation with CH_4_ and VFA production and is also a main reason for the decrease in VFA contents when we inhibited methanogenesis.

In contrast to fibrolytic bacteria, *Complylobacter* and *Prevotella* exhibited a negative correlation with gas, CH_4_, ruminal hydrogen balance, and VFAs (except the A/P ratio). This is mainly because these taxa are associated with nitrogen metabolism and the pentose phosphate pathway [90]. These findings are in line with earlier reports describing a lesser importance of *Prevotella* (as it explains only 14.9% of variability) in CH_4_ emissions [90].

Fibrolytic bacteria (including *Ruminococcus*), some genera from Clostridium, and Bacteriodetes showed a positive correlation with individual AA (except valine and cysteine) and total essential and non-essential AA contents. As we used a low-protein diet with 90% roughage, fiber was a major substrate available that favored fibrolytic bacterial abundance, leading to cellulolytic activity and subsequent synthesis of microbial AA and protein. That is why fibrolytic taxa showed a positive correlation with VFAs and ruminal AA contents.

## 5. Conclusions

Our study revealed no effect of methionine on methanogenesis, but a significant reduction in cumulative gas and CH_4_ production in response to nitrate was observed. The higher level of methionine (1.12%) exhibited a negative effect on ruminal AA contents (except cysteine and asparagine). The lower level of methionine showed no effect on ruminal AA contents (except asparagine, which increased significantly compared to the control group). However, nitrate supplementation significantly increased the total and individual AA contents (except cysteine). The bacterial genera *Prevotella* and *Proteobacteria* were identified as biomarker taxa in the nitrate group, while *Compylobactor* was found to be the genus most affected by methionine. Most abundant pathways revealed through metagenomic functional prediction were related to carbohydrate, amino acid, and energy metabolism. Our findings indicated that methionine could increase ruminal asparagine content and total protozoa and *Compylobactor* populations, while exhibiting no effect on CH_4_ and total gas production.

## Figures and Tables

**Table 1:** Chemical composition of the substrate (DM basis).

Ingredient	Content
Elephant grass (%)	90
Concentrate mixture (%) *	10
**Chemical Composition**	
Dry matter (%)	20
Crude protein (%)	9.03
Neutral detergent fiber (%)	75.34
Acid detergent fiber (%)	46.01
Ash (%)	9.72
Gross energy (kcal/kg DM)	4.69

* Concentrate mixture (corn 17.83%, wheat bran 7.51%, soybean meal 5.72%, limestone 0.5%, CaHPO_4_ 0.6%, NaHCO_3_ 0.8%, NaCl 0.7%, Premix1 0.34%). The additive premix provided the diet with the following (per kg of diet): VA 550,000 IU, VE 3000 IU, VD3 150,000 IU, 4.0 g Fe (as ferrous sulfate), 1.3 g Cu (as copper sulfate), 3.0 g Mn (as manganese sulfate), 6.0 g Zn (as zinc sulfate), 80 mg Co (as cobalt sulfate).

**Table 2:** Quantity of substrate and treatment used in each in vitro bottle.

	Control	M0 (0% Methionine)	M1 (0.28% Methionine)	M2 (1.12% Methionine)
Substrate	0.5 g	0.5 g	0.5 g	0.5 g
NaNO_3_	0	0.06 g	0.06 g	0.06 g
Methionine	0	0	0.0014 g	0.0056 g

**Table 3:** In vitro rumen fermentation parameters.

Parameter	Control	M0 (0%)	M1 (0.28%)	M2 (1.12%)	SEM	*p* Value
CH_4_ (mL/g DM)	25.69 ^a^	5.88 ^b^	5.99 ^b^	6.31 ^b^	0.601	0.001
Total gas (mL/g DM)	205.2 ^a^	110.8 ^b^	117.2 ^b^	113.2 ^b^	4.001	0.001
**Reductive hydrogen**						
H_2_ produced (mmol)	7.72 ^a^	6.26 ^b^	6.40 ^b^	6.25 ^b^	0.141	0.001
H_2_ utilized (mmol)	5.65 ^a^	2.99 ^b^	3.01 ^b^	3.05 ^b^	1.093	0.001
H_2_ recovery (%)	73.40 ^a^	47.88 ^c^	47.10 ^c^	48.88 ^c^	0.085	0.001

Values with different superscripts in the same row differ significantly.

**Table 4:** In vitro rumen fermentation parameters.

	Control	M0	M1	M2	SEM	*p* Value
pH	6.70 ^c^	6.84 ^a^	6.78 ^b^	6.80 ^a^	0.023	0.001
Acetate (mmole/L)	32.32	32.21	32.81	31.94	0.547	0.748
Propionate (mmole/L)	17.54 ^a^	15.01 ^b^	14.67 ^b^	15.03 ^b^	0.336	0.001
Isobutyrate (mmole/L)	1.10 ^a^	0.59 ^b^	0.58 ^b^	0.56 ^b^	0.021	0.001
Butyrate (mmole/L)	9.81 ^a^	5.17 ^b^	5.58 ^b^	5.27 ^b^	0.238	0.001
Isovalerate (mmole/L)	2.31 ^a^	1.35 ^b^	1.19 ^b^	1.30 ^b^	0.066	0.001
Valerate (mmole/L)	1.31 ^a^	0.80 ^b^	0.81 ^b^	0.77 ^b^	0.025	0.001
MCP (mg/mL)	3.43 ^a^	4.33 ^b^	4.49 ^b^	4.47 ^b^	0.093	0.001
NH_3_-N (mg/mL)	18.32	17.81	18.45	18.78	0.318	0.272
TVFA (mmole/L)	64.38 ^a^	55.13 ^b^	55.64 ^b^	54.87 ^b^	0.957	0.001
A/P	1.84 ^a^	2.17 ^b^	2.24 ^b^	2.13 ^b^	0.047	0.001

Values with different superscripts in the same row differ significantly.

**Table 5:** Amino acid profile in different treatment groups (ng/mL).

Amino Acid	Control	M0	M1	M2	SEM	*p* Value
Alanine	327.72 ^b^	753.56 ^a^	615.46 ^a^	296.67 ^b^	82.01	0.001
Valine	62.62 ^b^	121.60 ^ab^	141.98 ^a^	65.68 ^b^	19.73	0.014
Histidine	52.19 ^bc^	76.62 ^a^	60.15 ^ab^	35.86 ^c^	7.69	0.006
Arginine	30.89 ^b^	53.31 ^a^	49.89 ^a^	26.99 ^b^	5.57	0.003
Glycine	184.32 ^bc^	363.54 ^a^	303.09 ^ab^	136.81 ^c^	42.16	0.002
Glutamine	409.13 ^b^	822.82 ^a^	681.91 ^a^	370.95 ^b^	78.99	0.001
Glutamate	786.52 ^b^	1402.61 ^a^	1212.22 ^a^	652.38 ^b^	134.52	0.001
Proline	36.54 ^bc^	67.70 ^a^	61.17 ^ab^	23.97 ^c^	8.65	0.003
Leucine	151.82 ^b^	359.94 ^a^	296.21 ^a^	131.80 ^b^	43.08	0.001
Lysine	472.00 ^b^	970.89 ^a^	807.53 ^a^	431.42 ^b^	93.51	0.001
Methionine	96.22 ^b^	222.14 ^a^	192.11 ^a^	99.98 ^b^	23.19	0.001
Tryptophan	71.19 ^b^	127.54 ^a^	109.68 ^a^	61.12 ^b^	13.26	0.003
Phenylalanine	170.84 ^b^	355.42 ^a^	313.22 ^a^	153.91 ^b^	39.72	0.001
Threonine	166.87 ^bc^	358.22 ^a^	289.51 ^ab^	131.18 ^c^	43.19	0.002
Isoleucine	152.68 ^b^	357.16 ^a^	298.67 ^a^	133.66 ^b^	42.63	0.001
Tyrosine	45.99 ^b^	88.84 ^a^	84.05 ^a^	37.78 ^b^	10.24	0.001
Serine	188.18 ^b^	379.51 ^a^	322.97 ^a^	155.95 ^b^	41.06	0.001
Aspartic acid	141.08 ^b^	306.21 ^a^	254.98 ^a^	124.40 ^b^	35.05	0.002
Asparagine	8.187 ^b^	7.507 ^b^	19.431 ^a^	17.181 ^a^	2.553	0.003
Cysteine	57.86	69.48	70.70	65.89	3.90	0.105
Essential Amino acids ^1^	1396.44 ^bc^	2949.53 ^a^	2509.06 ^ab^	1244.60 ^c^	314.96	0.001
Non-Essential Amino acids ^2^	2216.41 ^bc^	4315.09 ^a^	3675.87 ^ab^	1908.97 ^c^	432.85	0.001
Total Amino Acids ^3^	3612.85 ^bc^	7264.62 ^a^	6184.93 ^ab^	3153.57 ^c^	747.33	0.001

Values with different superscripts in the same row differ significantly. ^1^ Histidine, isoleucine, leucine, lysine, methionine, phenylalanine, threonine, tryptophan, valine; ^2^ Alanine, arginine, glycine, glutamine, glutamate, proline, tyrosine, serine, aspartic acid, asparagine, cysteine; ^3^ Sum of essential and non-essential AA.

**Table 6:** Microbial populations (log10 copies per g of rumen contents).

Parameter	Control	M0	M1	M2	SEM	*p* Value
Bacteria	11.13 ^b^	11.44 ^a^	11.49 ^a^	11.47 ^a^	0.040	0.001
Fungi	8.40	8.64	8.70	8.57	0.087	0.109
Protozoa	7.31 ^b^	7.54 ^ab^	7.89 ^a^	7.66 ^ab^	0.125	0.020
Methanogens	9.32 ^b^	9.56 ^a^	9.60 ^a^	9.56 ^a^	0.046	0.001

Values with different superscripts in the same row differ significantly.

**Table 7:** Alpha diversity parameters across different treatment groups.

Parameter	Control	M0	M1	M2	SEM	*p* Value
Sobs	1740.20	1686.70	1638.90	1645.90	26.68	0.051
Shannon	6.08 ^a^	5.59 ^b^	5.36 ^b^	5.55 ^b^	0.07	0.001
Simpson	0.006 ^c^	0.021 ^b^	0.032 ^a^	0.022 ^ab^	0.002	0.001
ACE	2035.80	2043.20	2013.00	2018.50	25.93	0.841
Chao	2068.60	2063.10	2041.40	2053.60	29.78	0.927
Shannon evenness	0.82 ^a^	0.75 ^b^	0.72 ^b^	0.75 ^b^	0.01	0.001
Simpson evenness	0.10 ^a^	0.04 ^b^	0.02 ^b^	0.04 ^a^	0.01	0.001

Values with different superscripts in the same row differ significantly.

## Data Availability

The sequence data generated in this experiment (16SrRNA gene sequences) were deposited in SRA database of NCBI under Bioproject No. PRJNA646609 and SRA accession No. SRP272883.

## Supplementary Materials

The supplementary materials are available online at https://www.mdpi.com/article/10.3390/microorganisms9081717/s1. Table S1. PCR primers for real-time PCR assay; Table S2. Relative abundance of different phyla across treatment groups. Table S3. Relative abundance of different phyla across treatment groups.
