# Supplementary material for: Effect of Methionine Supplementation on Rumen Microbiota, Fermentation, and Amino Acid Metabolism in In Vitro Cultures Containing Nitrate
Source: Microorganisms. 2021 Aug 12;9(8):1717. doi: 10.3390/microorganisms9081717 (PMC8397988; doi:10.3390/microorganisms9081717)
Supplement: Supplementary file 1 [file microorganisms-09-01717-s001.zip › microorganisms-1285241-SI.pdf]

Supplementary Tables

Table S-1. PCR primers for real-time PCR assay

Target strain	Pre/post primer	Primer sequence	Amplification length (bp)
Total bacteria[1]	F	CGGCAACGACCGCAACCC	130
	R	CCATTGTAGCACGTGTGTAGCC	
Total fungi[1]	F	GAGGAAGTAAAAGTCGTAACAAGGTTTC	120
	R	CAAATTCACAAAGGGTAGGATGATT	
Total Protozoa[1]	F	GCTTTCGWTGGTAGTGTATT	223
	R	CTTGCCCTCYAATCGTWCT	
Total Methanogens[1]	F	TTCGGTGGATCDCARAGRGC	140
	R	GBARGTCGWAWCCGTAGAATCC	

F= forward; R= reverse.

[1]Ebeid et al., 2020 [24]

Table S2. Relative abundance of different phyla across treatment groups

Phylum	Control	M0	M1	M2	P value
Bacteroidetes	37.52	44.69	36.11	38.62	0.100
Firmicutes	41.76	31.67	36.62	37.80	0.019
Campilobacterota	0.09	10.18	15.62	11.45	0.001
Verrucomicrobiota	11.17	2.71	2.88	2.79	0.001
Proteobacteria	1.28	3.94	3.06	3.75	0.001
Spirochaetota	2.89	3.68	2.46	2.40	0.179
Actinobacteriota	1.17	0.68	0.85	0.91	0.179
Patescibacteria	1.04	0.57	0.58	0.64	0.009
Cyanobacteria	0.92	0.39	0.52	0.47	0.011

Table S-3. Relative abundance of different phyla across treatment groups

Species name	Control	M0	M1	M2	P value
Prevotella	3.78	19.03	14.50	13.85	0.001
Rikenellaceae_RC9_gut group	14.11	8.83	7.53	9.96	0.002
Campylobacter	0.02	10.02	15.48	11.31	0.001
norank_f_F082	10.28	4.45	4.28	4.90	0.001
norank_f__UCG-011	4.11	3.73	5.20	4.82	0.150
norank_f__norank_o__WCHB1-41	7.43	1.98	2.16	2.07	0.003
Succiniclasticum	2.71	2.27	3.36	3.45	0.567
NK4A214_group	2.69	2.34	2.92	3.06	0.440
Christensenellaceae_R-7_group	3.21	2.17	2.62	2.60	0.490
norank_f__Bacteroidales_UCG-001	1.14	2.86	2.41	2.24	0.003
norank_f__UCG-010	3.73	1.46	1.46	1.47	0.001
Butyrivibrio	1.21	1.60	2.17	2.36	0.073
norank_f__Muribaculaceae	1.47	1.76	1.40	1.48	0.972
Sphaerochaeta	1.82	1.68	1.01	0.84	0.013
Treponema	0.70	1.84	1.34	1.43	0.010
norank_f__Bacteroidales_RF16_group	0.81	1.22	1.51	1.43	0.005
Saccharofermentans	1.28	0.95	1.13	1.23	0.204
Ruminococcus	0.98	0.98	1.29	1.13	0.538
Lachnospiraceae_NK3A20_group	1.34	0.77	0.86	1.03	0.281
Prevotellaceae_UCG-003	0.74	1.26	0.73	0.88	0.109
